# Camera Color Correction for Cultural Heritage Preservation Based on Clustered Data

**DOI:** 10.3390/jimaging7070115

**Published:** 2021-07-13

**Authors:** Marco Trombini, Federica Ferraro, Emanuela Manfredi, Giovanni Petrillo, Silvana Dellepiane

**Affiliations:** 1 Department of Electrical, Electronics and Telecommunication Engineering and Naval Architecture, Università degli Studi di Genova, Via All’Opera Pia 11A, 16145 Genoa, Italy; marco.trombini@edu.unige.it (M.T.); federica.ferraro@edu.unige.it (F.F.); 2 Department of Chemistry and Industrial Chemistry, Università degli Studi di Genova, Via Dodecaneso 31, 16146 Genoa, Italy; giselle1861@yahoo.it (E.M.); giovanni.petrillo@unige.it (G.P.)

**Keywords:** color correction, chemical composition, camera characterization

## Abstract

Cultural heritage preservation is a crucial topic for our society. When dealing with fine art, color is a primary feature that encompasses much information related to the artwork’s conservation status and to the pigments’ composition. As an alternative to more sophisticated devices, the analysis and identification of color pigments may be addressed via a digital camera, i.e., a non-invasive, inexpensive, and portable tool for studying large surfaces. In the present study, we propose a new supervised approach to camera characterization based on clustered data in order to address the homoscedasticity of the acquired data. The experimental phase is conducted on a real pictorial dataset, where pigments are grouped according to their chromatic or chemical properties. The results show that such a procedure leads to better characterization with respect to state-of-the-art methods. In addition, the present study introduces a method to deal with organic pigments in a quantitative visual approach.

## 1. Introduction

Cultural heritage bears witness to life and history, provides an identity to nations, and represents an irreplaceable source of inspiration. Its importance from cultural, historical, and economic points of view is invaluable; thus, its preservation and valorization are crucial topics for our society. Natural aging and deterioration due to external agents endanger artworks such as paintings, sculptures, and architecture, and therefore diagnostic tools are needed for monitoring and preservation.

Monitoring historical artistic heritage consists of the evaluation of possible modifications of some characteristics of the object under observation. When it comes to a artwork or, more generally, a mono- or polychromatic surface, color is one of those characteristics, as it is easily perceivable by the human eye, allows one to distinguish an artwork, and provides information on the nature and status of an artwork.

Color analysis on artworks is generally performed via specific instruments such as colorimeters and spectrophotometers, both of which use sophisticated technologies to accurately and precisely quantify and define color, working in a device-independent color space as Commission Internationale de l’Éclairage (CIE) L*a*b* [1,2] This allows for objective assessment of color changes in order to monitor the state of the painting over time and appropriately plan periodic protection or restoration actions. Color studies of artworks could also make use of Infrared (IR) and Ultraviolet (UV) data (by means of, e.g., infrared reflectography, UV–Visible spectrophotometry, UV reflectance, etc.) or X-ray fluorescence spectroscopy (XRF) [3,4].

However, several drawbacks may limit the efficacy of such devices/methodologies. First, colorimeters and spectrophotometers give, as with XRF, pointwise measurements; thus, color studies on large areas require several time-consuming repetitions. Furthermore, even though spectrophotometers are defined as non-invasive devices [5], they must perfectly lean onto the artwork surface in order to exclude external light radiation, thus, risking ruining the painting. Finally, it is still rare nowadays that small laboratories are equipped with the abovementioned costly devices.

In order to address such issues, recent studies have proposed performing color monitoring through photographic documentation. Here, the necessary equipment is conceivably minimal and considerably cheaper, consisting of a professional digital camera and a photographic set with adequate lighting; then, the color data of each pixel of the selected area can be stored from a single photoshoot, limited only by illumination [6]. However, uncorrected digital data are not directly comparable, in terms of quantitative reliability, to the standard provided by the more specific spectrophotometric instrumentation. In addition, a well-defined procedure consisting of camera calibration, arrangement of lights, and positioning of the artwork, although necessary, is not sufficient per se for a correct comparison of digital data with colorimetric data. Finally, another major problem when using a digital camera for measuring color is that consumer-level sensors (either CCD or CMOS type) are typically uncalibrated. 

Therefore, camera characterization is needed, i.e., some specific digital image processing to transform raw color digital values into objective L*a*b* values equivalent to colorimetric measures. 

A common approach to minimize the difference between digital and colorimetric determinations relies on the application of a correction based on a least-squares regression to the uncalibrated digital data. Linear [7,8], nonlinear, and mixed [9] approaches have all been described in the literature. Regarding nonlinear regressions, one can mention polynomial regressions [10,11], neural networks (NNs) [12,13], and look-up tables [14]. In addition, the problem of different color spaces based on the acquisition device must be addressed. Indeed, camera data usually refer to RGB or sRGB color spaces. Several approaches have been proposed [15], such as linear or quadratic models, neural networks for L*a*b* regression starting from RGB values, and models requiring RGB data to be converted into XYZ values, which are then used to derive L*a*b* values, with and without a linearization of sRGB data via a gamma model. Gamma correction is also involved in the method [16].

Further aiming at minimization of the correction error, other features to be preserved may be considered. For instance, characterization should be robust across different illuminants and reflectance types, and across noise [17,18,19].

To achieve better results, the use of digital image processing techniques for camera characterization can also be combined with different disciplines. Indeed, a multidisciplinary approach allows one to deal with specific features related to the heterogeneity of the data under analysis. Therefore, in order to overcome the lack of homoscedasticity required to apply a single-step procedure, an innovative approach combining pattern recognition and image processing techniques with chemistry information is proposed here.

In the present study, 117 tiles from the database of diagnostic analyses of The Foundation Centre for Conservation and Restoration of Cultural Heritage “La Venaria Reale” (in collaboration with the National Institute of Metrological Research and Laboratorio Analisi Scientifiche of Regione Autonoma Valle d’Aosta) represent the basic dataset [3,20]. 

As proposed by the state-of-the-art literature, the methods of linear regression, polynomial regression, and NN [8,9,19] were initially applied herein to the whole dataset, but the resulting performances unfortunately proved to be not satisfactory. It is worthwhile mentioning that a preliminary camera calibration using the X-Rite ColorChecker Passport Photo failed to provide satisfactory results [9], as expected, due to the limited color content of such a color chart.

To understand the reason for such poor results, the work was adapted by conducting a closer investigation of the pigments’ characteristics and their corresponding statistical analysis in photographic images in order to overcome the significant lack of the homoscedasticity feature that is required for proper application of approaches in the literature, which work at a global level.

Consequently, from the perspective of optimizing the analysis, the original idea proposed herein is to apply state-of-the-art characterization methods to clusters of data rather than to the whole digital dataset, selected by means of two different criteria, i.e., the color and chemical properties of pigments. Regarding the latter, based on Kremer code, the main chemical element can be objectively defined for each pictorial layer analyzed.

To overcome the issue of a small amount of data and to find one-to-one correspondence between an image and colorimetric data, samples referring to the same tile are sorted by hue values, which provides coupled data and the use of supervised methods for precise and punctual color correction.

Thus, the application of several methods for camera characterization to numerous clusters of the base dataset is described hereinafter, in order to minimize the difference between digital and spectrophotometric quantitative color data, and therefore validate a handy diagnostic tool such as a digital camera for color determination. The best characterization approach results were achieved from a polynomial regression, while the predominant factor that affects the efficacy of the color correction could be found in the chemical composition, more precisely, in the nature of the central element. The best results were those splitting the data by chemical composition. In addition, the proposed method also proved to be effective with organic pigments, which could not be analyzed via standard approaches such as XRF; in fact, the latter has been employed to identify the presence of inorganic pigments, characterized by elements with an atomic number higher than 13. Instead, other non-invasive approaches for the study of organic pigments (usually referred to as “lakes”) include IR and Raman spectroscopy, but still require rather sophisticated instrumentation.

The considered approaches are briefly presented in Section 2, along with the dataset. Additionally, details on how data were collected and split into clusters and how camera images were used are provided.

Although a complete color analysis of artworks is also based on IR and UV data, the scope of the present study is to investigate how deep an analysis performed with traditional photographic data can be.

## 2. Materials and Methods

### 2.1. Background

Sensors’ responses to light distribution are clearly defined in the literature [21,22]. For the sake of clarity, let $I\left(\lambda \right)$ be the illuminant spectral power distribution falling on the surface patch (λ is the wavelength), and let $\gamma \left(\lambda \right)$ be the reflectance function of the material the object is made from (or that its surface is painted with), so that the spectral power distribution $P\left(\lambda \right)$ can be expressed as follows:

(1) $$P\left(\lambda \right)=I\left(\lambda \right)\gamma \left(\lambda \right)$$

where $P\left(\lambda \right)$ is the spectrum of the light that reaches the sensor and is associated with the corresponding pixels of the image.

Then, let $\sigma \left(\lambda \right)$ be the spectral filter function of the sensor, and define the sensor’s response to $P\left(\lambda \right)$ as follows:

(2) $$s=\int_{\lambda }^{}P\left(\lambda \right)\sigma \left(\lambda \right)d\lambda$$

As mentioned in the Introduction, in the present study, camera and colorimeter sensors are involved. Hence, hereinafter, whenever s refers to the camera, it will be referred to as $c_{m}$, while the colorimeter, s, will be referred to as $c_{l}$ (which will be the reference measurement). Specifically, based on the available data, $c_{m}$ is a three-dimensional vector $c_{m}={\left[R,G,B\right]}^{T}$, laying in the RGB color space. Similarly, the colorimeter response comes from the device-independent color space CIE L*a*b*, namely $c_{l}={\left[L,a,b\right]}^{T}$.

In order to perform an efficient correction on error-prone measurements of color changing, such as those deriving from commercial cameras, an optimal transformation f such that $c_{m}\overset{f}{\to }c_{l}$ must be found. In fact, the final value of such a correction is only an approximation of the real corresponding $c_{m}$ value, namely $f\left(c_{m}\right)=\hat{c_{l}}$, due to the different nature of the considered color spaces, to noise, estimation, and computation errors, etc. Some constraints can be added to improve the precision of the correction and are discussed later in the paper. The general requirement for the function f is to be error-minimizing, i.e.:

(3) $$f=argmin_{g}\overset{N}{\underset{i=1}{\sum }}||u\left(c_{l}\right)-u\left(g(c_{m}\right))||,\;$$

where *N* is the number of color triplets in the dataset, u is a color space transformation to ensure that $g\left(c_{m}\right)$ and $c_{l}$ refer to the same color space, and $\left|\left|\cdot \right|\right|$ is the norm. In the present manuscript, the considered norms will be the root mean squared error (Euclidean distance) and ΔE_00_ [23]. In addition, a similarity measure will also be involved, i.e., Pearson’s correlation coefficient.

In the following, since f is properly designed to correct $c_{m}$ to be more similar to $c_{l}$, hence, both $c_{l}$ and $f\left(c_{m}\right)$ are in the CIE L*a*b* color space, and u is assumed to be the identity function.

In general, methods in the literature are applied to the entire dataset. However, it appeared that no conditions for a single correction were present because of the non-homoscedasticity of the data. Hence, the methods were applied to clusters of tiles that could be determined according to some criterion. Here, this multi-cluster approach is based on either the chemical element or color, which is the major novelty of this study. 

In this paper, the dependance of the color on the predominant chemical composition as well as on its chromaticity is investigated. More specifically, let $C_{i}$ be the i-th cluster of color, based on either the chemical properties or the chromaticity. The purpose is to find many functions $f_{i}$, one for each cluster, which, of course, depends on the cluster that the input color belongs to:

(4) $$c_{m}\in C_{i}\;⟹\hat{c_{l}}=f_{i}\left(c_{m}\right)=f(c_{m}|C_{i})$$

### 2.2. Instrumentation

According to the CIE standard definition [24], reference measurements were made using a Konica Minolta CM2600d spectrophotometer (Konica Minolta, Ramsey, NJ, USA) [25] with the following setup: standard observer at 10°, illuminant D65, and acquisition SCI. Five measurements were acquired for each pictorial layer.

Photographic image data were acquired with a Lumix DMC-FZ200 camera (Panasonic, Osaka, Japan). The following image acquisition setup was used: The camera was placed vertically at 46.5 cm from the samples. The angle between the axis of the lens and the sources of illumination was approximately 45°. Illumination was achieved with two Natural Daylight 23 W fluorescent lights (OSRAM, Munich, Germany), color temperature 6500 K, reproducing the standard D65 illuminant. The photos were shot in a dark room. The settings of the camera are summarized in Table 1.

### 2.3. Dataset

**Figure 1:**
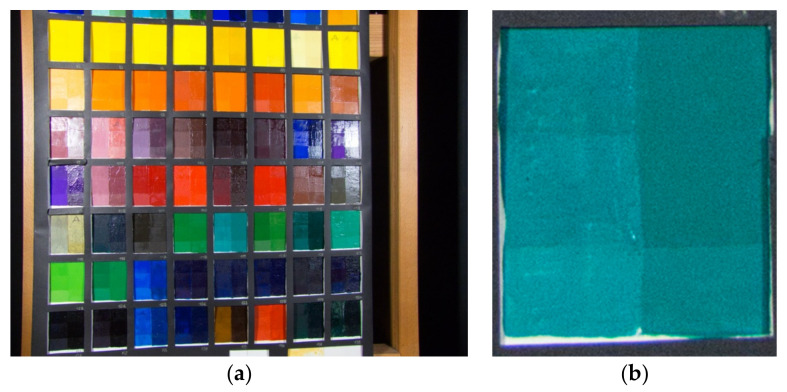
(**a**) On the left, a picture of a table with a collection of colored tiles from The Foundation Centre for Conservation and Restoration of Cultural Heritage “La Venaria Reale”; (**b**) on the right, a single tile. The reader may notice the presence of two columns and three rows. The red box indicates the area considered for this study.

As previously mentioned, the dataset of the present study consisted of 117 tiles from the database of diagnostic analyses of La Venaria Reale [20]. A picture for each tile was taken to enable analysis. Figure 1a shows an example of a photographic picture of the tables from Venaria.

**Figure 2:**
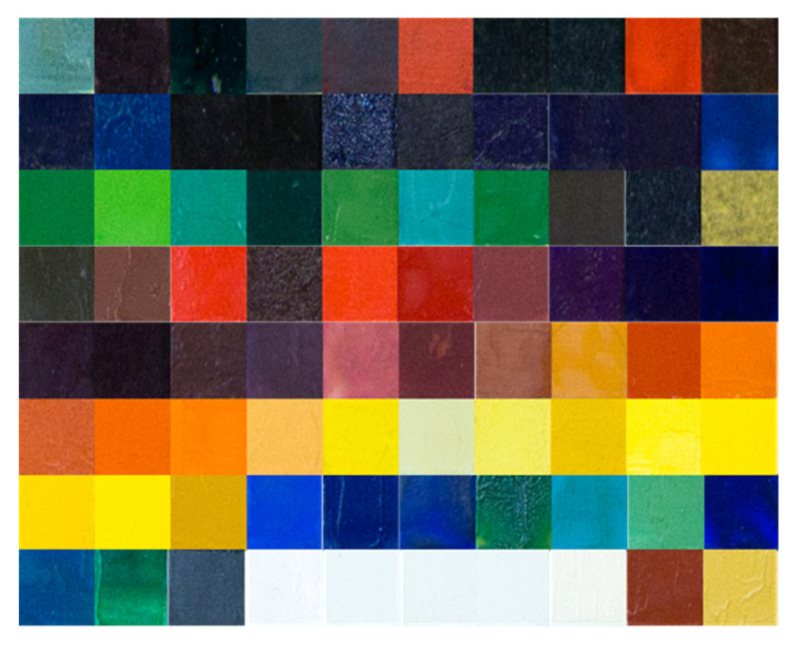
A subset of tile samples involved in the present study.

In the table, each pigment (Figure 1b) is presented in a mixture with two binders: polyvinyl acetate (PVAc) (column on the left) and linseed oil (column on the right). Then, the painted surface is divided into 3 rows. The first two present 2 different finishings: terpene resin (stripe on the top) and acrylic resin (middle stripe), while the third one is unprotected. For the present study, only the unprotected and the linseed oil sectors were taken into consideration (the red box in Figure 1b), because the linseed oil technique is the one most used by painters since the 15th century. The central portion of the camera acquisition was considered in order to avoid specularity and saturation problems. Figure 2 shows some of the selected parts of tiles involved in the study.

To address the local color inhomogeneity of tiles, the characterization was performed by taking into consideration five measurements via the colorimeter and five RGB triplets extracted from the pictures in order to create paired couples and to develop a robust supervised color correction. 

Specifically, pixels from each tile were sorted by hue (in ascending order). Then, five triplets were extracted, namely the first one (i.e., the one with minimal hue), the last one (i.e., the one with maximal hue), and the ones corresponding to the 25th, 50th, and 75th percentiles. This was done to obtain as many samples as possible for the reference dataset.

### 2.4. Linear Regression

The method consists of estimating the L*, a*, and b* values separately via linear regression. In particular, let $\left(\alpha_{L},\beta_{L}\right)$, $\left(\alpha_{a},\beta_{a}\right)$, and $\left(\alpha_{b},\beta_{b}\right)$ be the regression coefficients for L*, a*, and b*, respectively, so that the estimated values are $L_{e}=\alpha_{L}L_{l}+\beta_{L}$, $a_{e}=\alpha_{a}a_{l}+\beta_{a}$, and $b_{e}=\alpha_{b}b_{l}+\beta_{b}$, where $L_{l}$, $a_{l}$, and $b_{l}$ are the colorimeter values. To find the best characterization of the camera data with $\hat{L_{m}},$ $\hat{a_{m},}$ and $\hat{b_{m}}$, the constraints $L_{m}\to \hat{L_{m}}≅L_{l}$, $a_{m}\to \hat{a_{m}}≅a_{l}$, and $b_{m}\to \hat{b_{m}}≅b_{l}$ are added, yielding the following:

(5) $$\hat{L_{m}}=\frac{L_{e}-\beta_{L}}{\alpha_{L}},\;\hat{a_{m}}=\frac{a_{e}-\beta_{a}}{\alpha_{a}},\;\hat{b_{m}}=\frac{b_{e}-\beta_{b}}{\alpha_{b}}$$

### 2.5. Polynomial Regression

The polynomial regression approach consists of mapping a polynomial expansion of the device RGB values to estimated L*a*b*. In the following, the polynomial $P_{8}$ was used:

(6) $$P_{8}=\left[R,G,B,RG,RB,GB,RGB,1\right],$$

The corrected L*a*b* triplet $\hat{c_{l}}$ is obtained via the following equation:

(7) $$\hat{c_{l}}=MP_{8},$$

where M is the 3×8 tranformation matrix, which is derived via a pseudo-inversion procedure as in [11].

### 2.6. Hue-Plane-Preserving Camera Characterization—Weighted Constrained Matrixing Method

The Hue-Plane-Preserving Camera Characterization—Weighted Constrained Matrixing (HPPCC-WCM) method [19] is aimed at ensuring that the characterization preserves the hue plane and minimizes error. Starting from the camera data, the transformation matrix is defined in function of the device hue angle $\varphi_{m}$ and of the parameter p referring to the order of the transformation, as follows:

(8) $$M\left(\varphi_{m},p\right)=\frac{1}{\sigma }\sum_{i=1}^{N}{\left(\pi -\Delta \varphi_{i}\right)}^{p}M_{i},$$

where N is the number of training coupled colorimeter–camera data ($c_{l},c_{m}$), $M_{i}$ is the transforming matrix $c_{m,i}=M_{i}c_{l,i}$, $\Delta \varphi_{i}=min\left(|\varphi_{m}-\varphi_{i}\left|,2\pi -|\varphi_{m}-\varphi_{i}\right|\right)$, with $\varphi_{i}$ being the *i*-th training color hue angle, and $\sigma =\overset{N}{\underset{i=1}{\sum }}{\left(\pi -\Delta \varphi_{i}\right)}^{p}$.

To sum up, the color correction here proposed is as follows:

(9) $$\hat{c_{l}}=M\left(\varphi_{m},p\right)c_{m}$$

### 2.7. Data Grouping

To avoid the application of each method in a global way, the dataset under analysis was clustered according to two different criteria, i.e., according to chromatic appearance and the chemical composition, with reference to the central metal atom. Table A1 in Appendix A shows the available pigments and relevant features (pigment name and color, chemical composition, chemical cluster, and chromatic cluster). Regarding the chromatic appearance, five classes were subjectively identified. Conversely, regarding the chemical composition, Kremer code [26] was objectively considered. The clusters and relevant numbers of tiles are summarized in Table 2.

Some considerations regarding this clustering are made in the following.

In general, three phases drove the choice of the different chemical clusters. Firstly, three major classes were considered referring to the elements most spread in the dataset: iron, lead, and copper (Phase 1). 

Then, by looking at the copper class, it was found that some tiles were organic lakes, generating the idea that this clustering method could also be effectively applied to organic dyestuff. Accordingly, the clusters “copper (organic)” and “organic” (collecting all the lakes in the dataset) were considered (Phase 2). 

Finally, a mixed class was also considered, characterized by the presence of either iron, manganese, or cobalt, i.e., vicinal transition metals with very similar electronic properties (Phase 3).

Regarding the chromatic clusters, the gray cluster collects pigments with similar R, G, and B values (thus also including black and white pigments).

In Table A1, one can notice that the color grouping of some pigments differs from the chromatic class to which they belong, according to the closest color perception. For example, tile number 57, despite being visually brown/violet, also has shades of red given by its chemical description provided by Kremer, which identifies it as a red pigment. 

### 2.8. Proposed Method

The proposed approach involves a combination of the aforementioned procedures. The data grouping procedure splits the dataset into clusters, which are homogeneous in terms of either color or chemical properties. The color correction methods are independently applied to each cluster. Recall that colorimetric and camera data are precisely coupled by hue, as specified in Section 2.3. Altogether, this leads to an adaptive color correction method. 

## 3. Results

The color correction process was assessed via a five-fold cross-validation approach. The effectiveness of the procedure was evaluated on the grounds of statistical parameters such as Pearson’s correlation coefficient and the three measures of color distance. The root mean squared error in the L*, a*, and b* parameters (RMS) and the related color distance measure expressed in color units, according to the formula $\Delta =\sqrt{RMS{\left(L\right)}^{2}+RMS{\left(a\right)}^{2}+RMS{\left(b\right)}^{2}}$ [23], represent traditional metrics. The ΔE_00_ distance, officially adopted in 2001 as the new CIE color difference equation, improves the performance on blue and gray colors thanks to an interactive term between chroma and hue differences and a scaling factor for the CIELAB a* scale, respectively [27]. The latter is implemented, here, according to the CIEDE2000 formula [21] (MATLAB implementation [28]). 

The relevant values in Table 3, Table 4, Table 5, Table 6, Table 7, Table 8, Table 9, Table 10, Table 11, Table 12 and Table 13 are the means of the five attempts performed during the cross-validation. In addition, the error associated with each value is specified in brackets. It was computed as the semi-difference between the maximum and minimum values in the five measurements, and it assesses the robustness of the k-fold procedure.

**Figure 3:**
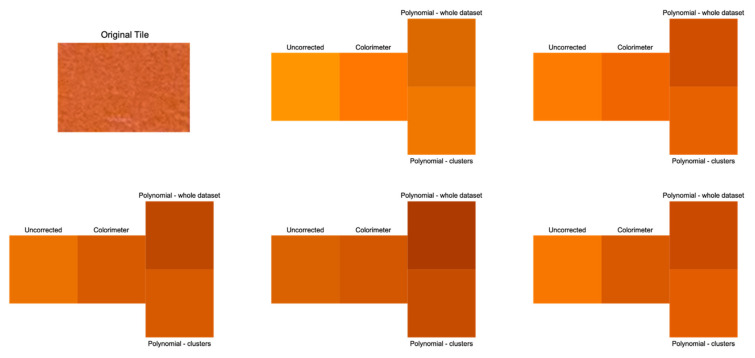
Images depicting an example of color correction by means of the polynomial regression method.

Some examples of color correction applied to the pigments are reported in Figure 3. All five measurements extracted from the considered tile are shown, coupled according to the described approach. Each visualization depicts the uncalibrated color values, the colorimeter data, and the correction when the polynomial regression characterization was trained on the whole dataset and on the specific cluster. The reader may notice the improvement in the visual rendering when dealing with clustered data by chromatic properties.

## 4. Discussion

First, the obtained results are discussed in terms of the values of the metric considered. Then, the importance of the preliminary cluster analysis is highlighted, with observations mainly relevant to the two clustering procedures. To conclude, possible applications of the proposed pipeline are disclosed, along with the limitations of the present research and foreseeable future developments.

### 4.1. Discussing the Considered Indexes’ Values 

Table 3, referring to the application of the methods to the whole dataset, shows a strong agreement among the traditional metrics of correlation, the RMS on the L*, a*, and b* parameters, and the color distance Δ.

In general, for both the whole dataset and the different selected clusters, the characterization method that produced the best color correction was polynomial regression, which was always able to improve similarity with colorimetric data as compared with uncalibrated data. Linear regression dramatically worsened the result as compared with the original data, as did the HPPCC-WCM method on most of the indexes. However, even though polynomial regression always showed improvements, according to the ΔE_00_ distance, the HPPCC-WCM method outperformed the others since both the method and the metric rely on the more recent CIE standards, facing some drawbacks of the traditional standards.

Table 4, Table 5, Table 6 and Table 7 confirm the best performances of polynomial regression, which improved uncalibrated data on all clusters, even in the challenging case of copper (organic), where the acquired colorimetric and photographic L parameters showed a strong misalignment. 

Table 8 gives further evidence that the ΔE_00_ metric can solve some problems of traditional colorimetry as, except for the lead cluster, it gives better improvements. Additional results reported in Table 11 show that the blue, green, and, to a lesser extent, the gray clusters might benefit from hue preservation and the new metric, as declared in the new standard scope.

The Pearson coefficients were already high for the whole dataset; hence, the improvement obtained by clustering was less relevant for this index. Conversely, by taking into consideration RMS, ΔE_00_, and Δ, the improvement when passing from the global correction to the cluster-based correction was significant, as they both decreased when focusing on chemical and chromatic clusters. In fact, the expression of the prediction error in terms of color units is only intended to evaluate the human perception of the correction; indeed, recall that if the error is approximately less than 2.2 color units, then, the difference is considered to be imperceptible to the human eye. It is worthwhile noting the improvement in this index, which decreased from a mean value across classes of 27.56 (Table 12, “uncalibrated”) to 13.95 with respect to the chemical clusters (Table 12, “polynomial regression”), and from a mean value across classes of 24.14 (Table 13, “uncalibrated”) to 13.26 with respect to the chromatic clusters (Table 13, “polynomial regression”). In such a case, clustering based on the chemical components is the most effective procedure, i.e., the one producing the lowest error. It is expected that more sophisticated algorithms, which could be investigated in future developments of the present study, would lead to an even lower color unit error.

### 4.2. The Significance of the Clustering Procedure

Splitting the dataset into clusters led to a better color correction for both splitting criteria (chromatism or chemical composition). The efficacy of clustering can be appreciated by comparing the value of, for example, the Δ index for the whole dataset (Table 3, 16.60 after polynomial regression characterization, with a 32% decrease with respect to the value for the uncalibrated data) with the values for the single clusters in Table 12 and Table 13, for example, for the “lead” cluster, the value is 9.67, with a 54% decrease after characterization. Therefore, one can infer that the clustering procedure effectively addresses the homoscedasticity of the data. Indeed, the major contribution of the present study is the efficiency of the coupling between clustering and application of some state-of-the-art color correction methods. In addition, it is worth stressing that, although one might expect better results and a more effective color correction from chromatic clusters, the best correction was provided by chemical clusters. This is likely due to the objectivity of the chemical component criterion for defining clusters, while chromatic properties are more dependent on human perception, thus, leading to less homogeneous classes. 

To stress once more the novelty of the present study, to the best of our knowledge, such an approach as well as the results relevant to the different clustering criteria are unprecedented. 

### 4.3. Chromatic Clusters

As outlined above, clustering by chromatism seems less effective for color correction purposes. The perceived colors driving the selection of the chromatic clustering depend, to some extent, on the observer, and therefore are subjective. In addition, several color shades are present, which may lead to heterogeneous classes. Of course, having more samples for each tile would allow one to split the data into more classes, each being characterized by a closer chromatic similarity; as a result, the training phase would benefit, thus, conceivably leading to better color correction.

The correction provided by the polynomial regression method on the coordinates of the Lab color space suggests some additional considerations, recalling that “L” represents the perceptual lightness, while “a” and “b” refer to the four colors in the opposite component model of human vision, i.e., red, green, blue, and yellow.

The most improved coordinate was L, meaning that this procedure addresses the problems in terms of the lightness sensitivity of photographic data. Without correction, the error is so high that the observer perceives a consistently different color with respect to the colorimetric data (see Figure 3).

Regarding the coordinates “a” and “b”, it is interesting to consider the chromatic class of gray. The values in this class are supposed to be similar, and so the difference between colorimeter and photographic data should also be similar. However, by looking at the RMS index (Table 10), we found that the difference between colorimeter and photographic data was much higher for “b” than for “a”. Conversely, once the values were corrected with polynomial regression, the errors were similar, thus, suggesting that the procedure is useful to address some imbalance for the gray class.

### 4.4. Chemical Clusters

The criterion based on chemical composition is more univocal, an aspect that surely contributes, in general, to the results being more similar as well as rewarding across the considered classes.

In particular, the RMS index mirrors a gratifying, effective color correction for the main elemental clusters (lead, iron, and copper, see Table 6) after polynomial regression characterization.

Regarding the additional elemental classes reported in Table 2, particular attention must be paid to the copper-based samples. In fact, the “copper” cluster of Table 2 (16 samples) also included nine organic samples, where copper was the metal cation of an organic salt, which made up the selected subcluster defined as “copper (organic)”. In terms of the RMS index, both the “copper” cluster and the subcluster performed extremely well (Table 6 and Table 7, respectively) as far as the “L” component was concerned, while the components “a” and “b” did not seem to be significantly corrected for the subcluster by the characterization method of choice. Nonetheless, we paid attention to the consistent number of tiles containing organic pictorial matter (either organic dyes or metal salts of organic acids); satisfactorily enough, the rather crowded (34 samples of lakes) “organic dyes and salts” cluster responded positively to the polynomial regression characterization (as compared with the values of the RMS index in Table 7 or of the ∆ index in Table 12) or to the HPPCC-WCM treatment (as compared with the value of the ∆E_00_ index in Table 8). 

The performance provided by the cluster of lakes represents, in our opinion, a further original and very interesting aspect of the camera characterization procedure herein. This is because the identification and study of organic matter on pictorial artworks cannot be achieved by means of XRF, a non-invasive technique that is widely applied in the presence of pigments containing heavy metals, but which fails to detect organic dyestuff because C, N, and O atoms are too light. Instead, the current approach based on the correction of digital data grouped in elemental clusters does not depend on the atomic weight, and thus opens very appealing perspectives as to the analysis of lakes. Developments and applications to study cases are necessary to sustain this hypothesis. 

A first hypothesis about the reason why elemental clustering is a good approach is suggested by the results of the analysis on the mixed “iron, manganese, and cobalt” cluster. In fact, these three elements are transition metals adjacent in the periodic table, whose electronic configuration differs only for the number of electrons at the internal level, with the external one being identical for all three. The good results obtained for such a mixed class may mean that the proposed approach is sensitive to the outermost electronic level. Of course, more experimental trials are needed to validate the hypothesis, particularly by selecting other mixed clusters responding to the same characteristics.

### 4.5. A Possible Usage of the System for the Programming of Restoration Actions

A main concern about cultural heritage is the preservation of artwork for future generations. Of course, artworks, whatever the typology, inevitably tend to change or degrade with time due to several different causes, and restoration campaigns must be conducted whenever necessary. As far as pictorial artworks are concerned, color is surely the main sentinel to be observed in order to decide what actions to take. A handy and low-cost tool such as a digital camera would be optimal for frequent periodic control on artworks, as well as on large surfaces. In this way, time-dependent data describing the state of the paintings could be easily collected and analyzed preliminarily to further deepen more sophisticated analyses, if necessary, prior to a restoration action. 

To this end, repeated periodical collections of data are necessary to verify the feasibility of selecting a parameter as a valid index of color deterioration. While elemental clustering has proven optimal for the identification of color, it could be foreseen that chromatic clustering would be best to handle the fading/deterioration of color with time. Of course, at the present time, this is only a conjecture to be verified in the future as a compulsory development of the present study.

### 4.6. Limitations and Future Developments

First, the amount of available data needs to be increased, as it is supposed that it would lead to better correction, at least on statistical grounds. 

Of course, the present study cannot be limited to “theory”; in addition to the desirable significance of the method outlined in the previous paragraph, a main interest would be the application of the training to real cases in order to perform identification and study of the pictorial layers of an unknown composition. Thus, once the chemical clusters have been characterized, one can consider an “unknown” painting and focus on a particular area. If such an area fits a particular cluster, i.e., proper color correction is obtained by considering the parameters for that class, then it would mean that the relevant chemical elements are present in the considered area. 

A continuing collaboration with the laboratories of “La Venaria Reale” and contacts with museums or galleries would surely satisfy both the outlined forms of progress and enable the development of a novel machine-learning-based approach, which is presently hampered by the limited size of the available dataset.

## 5. Conclusions

A dataset of digital camera photographs and of colorimetric measurements on 117 tiles from the database of diagnostic analyses of The Foundation Centre for Conservation and Restoration of Cultural Heritage “La Venaria Reale” was collected and analyzed with the aim of minimizing the difference between digital and spectrophotometric quantitative color data, from the perspective of validating a handy diagnostic tool such as a digital camera for quantitative color determination. 

To address the homoscedasticity of the data acquired, the current study proposed a supervised approach to camera characterization and color correction based on clustered data. To this end, within the dataset, samples were grouped into clusters based on either the chromatic or the chemical properties of the pigments.

Among the different approaches studied in the present study, a polynomial regression obtained the best results with both of the proposed clustering criteria. Thus, while the correlation between characterized photographic data and colorimetric data remains high when considering both the entire dataset and the single clusters, in the latter case, notable improvements can be seen in the three parameters considered to test the efficacy of the characterization (i.e., RMS, ΔE_00_, and Δ). The central thesis that the piecewise method improves prediction accuracy was supported by numerical evaluations, even though, in absolute terms, the results were short of an error low enough to be imperceptible to a human expert.

In future studies, the aim could be to extend the dataset, for example, by developing the collaboration with La Venaria Reale. Of course, increasing the dataset would allow one to define new or more densely populated clusters, and therefore study the chemical and chromatic properties of the pigments in more detail, hopefully confirming the hypotheses above. A larger dataset may substantially improve the error, and therefore achieve imperceptible differences between the acquired data and the corrected data.

Furthermore, different approaches could be investigated, no longer based on the mean value of the colorimetric data, but rather looking for other significant parameters to perform the analysis. Additionally, further applications of the proposed approach are being investigated, such as applying it for characterizing the chemical composition of unknown artworks by leveraging the photographic data.

## Figures and Tables

**Table 1:** Camera setup.

Variable	Value
Focal distance	4 mm
Flash	Off
ISO speed	400
Operation mode	Manual
Exposure time	1/60 s
Quality	Raw
f-Number	f/3.2

**Table 2:** Number of tiles for each selected cluster.

Red	33	Iron	23
Green	16	Lead	10
Blue	31	Copper	16
Yellow	23	Copper (organic)	9
Gray	14	Organic dyes and salts	34
		Iron, manganese, and cobalt	31
		Other	36

**Table 3:** Evaluation of the considered methods for the whole dataset. The bold font highlights the best values throughout.

	Pearson’s Coefficient			RMS		
	L	a	b	L	a	b
Uncalibrated	0.95 (0.03)	0.85 (0.02)	0.95 (0.02)	13.52 (3.94)	13.26 (4.28)	15.22 (5.12)
Linear regression	0.80 (0.03)	−0.16 (0.01)	−0.34 (0.02)	106.70 (9.44)	126.18 (18.11)	98.22 (11.26)
Polynomial regression	**0.95 (0.02)**	**0.91 (0.01)**	**0.96 (0.02)**	**8.17 (1.57)**	**9.89 (2.08)**	**10.53 (1.88)**
HPPCC-WCM	0.94 (0.03)	0.87 (0.03)	0.44 (0.02)	38.30 (9.44)	46.83 (8.11)	90.74 (11.91)
		**ΔE_00_**			**Δ**	
Uncalibrated		127.31 (10.5)			24.30 (4.16)	
Linear regression		140.13 (14.16)			192.23 (14.10)	
Polynomial regression		101.26 (8.93)			**16.60 (2.94)**	
HPPCC-WCM		**53.52 (7.18)**			109.06 (9.87)	

**Table 4:** Pearson’s correlation coefficients of the considered methods for the major chemical clusters (Phase 1). The bold font highlights the best values throughout.

	**Lead**			**Iron**		
	**L**	**a**	**b**	**L**	**a**	**b**
Uncalibrated	0.92 (0.03)	0.93 (0.03)	**0.97 (0.02)**	0.76 (0.02)	0.92 (0.02)	0.90 (0.02)
Linear regression	0.59 (0.03)	−0.67 (0.03)	−0.87 (0.03)	0.87 (0.03)	0.16 (0.03)	−0.28 (0.02)
Polynomial regression	**0.92 (0.02)**	**0.98 (0.02)**	**0.97 (0.02)**	**0.91 (0.02)**	**0.93 (0.02)**	**0.94 (0.01)**
HPPCC-WCM	0.66 (0.02)	0.90 (0.03)	0.84 (0.01)	0.85 (0.03)	0.76 (0.03)	0.25 (0.02)
	**Copper**					
	**L**	**a**	**b**			
Uncalibrated	0.89 (0.03)	0.51 (0.03)	0.78 (0.03)			
Linear regression	0.24 (0.01)	−0.16 (0.02)	0.02 (0.03)			
Polynomial regression	**0.91 (0.01)**	**0.87 (0.03)**	**0.92 (0.01)**			
HPPCC-WCM	0.66 (0.03)	0.84 (0.02)	0.81 (0.01)			

**Table 5:** Pearson’s correlation coefficients of the considered methods for the other chemical clusters (Phase 2 and Phase 3). The bold font highlights the best values throughout.

	**Copper (Organic)**			**Organic**		
	**L**	**a**	**b**	**L**	**a**	**b**
Uncalibrated	−0.31 (0.02)	0.75 (0.04)	0.66 (0.03)	0.91 (0.03)	0.88 (0.02)	0.90 (0.02)
Linear regression	−0.52 (0.02)	−0.04 (0.01)	−0.52 (0.03)	0.89 (0.03)	−0.11 (0.04)	−0.32 (0.03)
Polynomial regression	**0.77 (0.03)**	**0.90 (0.02)**	**0.92 (0.02)**	**0.97 (0.02)**	**0.91 (0.04)**	**0.92 (0.03)**
HPPCC-WCM	0.23 (0.02)	−0.43 (0.04)	0.22 (0.03)	0.92 (0.01)	0.78 (0.03)	−0.53 (0.05)
	**Iron + Mn + Co**					
	**L**	**a**	**b**			
Uncalibrated	0.77 (0.04)	0.93 (0.03)	0.90 (0.03)			
Linear regression	0.44 (0.03)	−0.56 (0.03)	−0.39 (0.02)			
Polynomial regression	**0.82 (0.02)**	**0.93 (0.02)**	**0.91 (0.02)**			
HPPCC-WCM	0.65 (0.03)	0.59 (0.03)	0.09 (0.03)			

**Table 6:** RMS of the considered methods for the major chemical clusters (Phase 1). The bold font highlights the best values throughout.

	**Lead**			**Iron**		
	**L**	**a**	**b**	**L**	**a**	**b**
Uncalibrated	12.46 (2.49)	9.96 (2.33)	13.87 (2.92)	14.02 (1.91)	5.98 (1.4)	13.91 (1.74)
Linear regression	156.97 (9.88)	198.51 (8.91)	143.58 (8.56)	81.13 (6.5)	58.64 (5.19)	51.34 (7.92)
Polynomial regression	**5.29 (2.03)**	**4.25 (2.94)**	**6.89 (0.8)**	**5.22 (2.53)**	**4.04 (1.97)**	**4.89 (1.69)**
HPPCC-WCM	35.59 (7.37)	44.91 (6.39)	123.48 (5.89)	82.81 (6.95)	122.67 (5.48)	152.47 (5.76)
	**Copper**					
	**L**	**a**	**b**			
Uncalibrated	12.42 (2.98)	19.45 (3.89)	14.97 (3.98)			
Linear regression	39.11 (7.13)	136.14 (6.89)	899.47 (44.13)			
Polynomial regression	**5.21 (1.42)**	**8.79 (3.03)**	**5.92 (1.78)**			
HPPCC-WCM	147.46 (14.71)	91.33 (6.86)	116.78 (12.03)			

**Table 7:** RMS of the considered methods for the other chemical clusters (Phase 2 and Phase 3). The bold font highlights the best values throughout.

	**Copper (Organic)**			**Organic**		
	**L**	**a**	**b**	**L**	**a**	**b**
Uncalibrated	13.07 (3.04)	10.44 (3.29)	12.02 (4.21)	17.77 (4.28)	23.12 (5.82)	21.38 (6.92)
Linear regression	31.48 (6.92)	166.89 (22.12)	66.34 (2.96)	164.24 (12.98)	231.84 (12.95)	89.29 (5.98)
Polynomial egression	**3.38 (0.24)**	**9.92 (2.31)**	**12.34 (4.32)**	**6.14 (3.07)**	**4.96 (0.45)**	**10.33 (1.89)**
HPPCC-WCM	142.29 (14.56)	113.95 (8.22)	125.13 (16.21)	39.34 (5.89)	87.43 (18.19)	194.90 (23.67)
	**Iron + Mn + Co**					
	**L**	**a**	**b**			
Uncalibrated	15.72 (2.68)	7.44 (2.03)	14.49 (2.23)			
Linear regression	73.15 (9.86)	79.74 (9.65)	73.92 (6.73)			
Polynomial regression	**7.47 (2.33)**	**6.83 (1.12)**	**7.39 (2.11)**			
HPPCC-WCM	89.09 (7)	109.32 (13.13)	132.52 (16.18)			

**Table 8:** ΔE_00_ of the considered methods for the chemical clusters. The bold font highlights the best values throughout.

	Lead	Iron	Copper	Copper (Organic)	Organic	Iron + Mn + Co
Uncalibrated	47.35 (9.68)	152.59 (13.72)	336.47 (24.68)	129.96 (7.34)	166.31 (29.76)	138.61 (12.91)
Linear regression	136.43 (13.8)	135.49 (14.25)	149.02 (18.32)	176.22 (16.43)	172.15 (32.94)	142.90 (19.94)
Polynomial regression	**9.78 (3.18)**	126.50 (10.18)	95.24 (8.58)	111.38 (13.23)	113.29 (22.63)	93.38 (8.22)
HPPCC-WCM	46.87 (5.97)	**79.42 (6.38)**	**68.48 (3.69)**	**82.11 (9.56)**	**78.62 (9.35)**	**83.09 (5.63)**

**Table 9:** Pearson’s correlation coefficients of the considered methods for the chromatic clusters. The bold font highlights the best values throughout.

	**Red**			**Green**		
	**L**	**a**	**b**	**L**	**a**	**b**
Uncalibrated	0.86 (0.03)	0.90 (0.03)	0.92 (0.02)	0.89 (0.03)	0.70 (0.02)	0.84 (0.02)
Linear regression	0.86 (0.04)	0.13 (0.03)	−0.60 (0.03)	0.32 (0.03)	−0.35 (0.04)	−0.15 (0.02)
Polynomial regression	**0.92 (0.02)**	**0.91 (0.01)**	**0.94 (0.03)**	**0.91 (0.01)**	**0.84 (0.02)**	**0.86 (0.01)**
HPPCC-WCM	0.91 (0.02)	0.47 (0.02)	−0.30 (0.01)	0.54 (0.03)	0.83 (0.02)	0.74 (0.01)
	**Blue**			**Yellow**		
	**L**	**a**	**b**	**L**	**a**	**b**
Uncalibrated	0.89 (0.03)	0.38 (0.03)	0.84 (0.02)	0.87 (0.02)	0.35 (0.03)	0.81 (0.02)
Linear regression	0.74 (0.02)	−0.33 (0.02)	0.04 (0.02)	0.39 (0.02)	−0.43 (0.01)	−0.71 (0.03)
Polynomial regression	**0.92 (0.02)**	0.73 (0.02)	**0.89 (0.01)**	**0.90 (0.03)**	**0.87 (0.03)**	**0.92 (0.02)**
HPPCC-WCM	0.73 (0.02)	**0.87 (0.01)**	0.75 (0.02)	0.78 (0.01)	0.65 (0.01)	−0.13 (0.02)
	**Gray**					
	**L**	**a**	**b**			
Uncalibrated	0.99 (0.01)	0.74 (0.01)	0.76 (0.01)			
Linear regression	0.98 (0.02)	0.18 (0.03)	0.10 (0.02)			
Polynomial regression	**0.99 (0.01)**	0.88 (0.01)	0.75 (0.01)			
HPPCC-WCM	0.98 (0.02)	**0.99 (0.01)**	**0.95 (0.02)**			

**Table 10:** RMS of the considered methods for the chromatic clusters. The bold font highlights the best values throughout.

	**Red**			**Green**		
	**L**	**a**	**b**	**L**	**a**	**b**
Uncalibrated	14.72 (2.58)	9.70 (1.56)	14.68 (2.01)	13.58 (2.09)	14.32 (3.23)	12.77 (2.98)
Linear regression	138.58 (7.55)	62.36 (5.79)	53.43 (6.69)	47.86 (7.36)	142.85 (11.81)	80.63 (6.40)
Polynomial regression	**6.64 (1.73)**	**6.31 (0.82)**	**9.42 (1.72)**	**6.72 (1.23)**	**10.28 (2.34)**	**8.23 (0.82)**
HPPCC-WCM	36.15 (2.68)	129.90 (7.76)	176.77 (11.24)	118.15 (15.73)	69.96 (4.61)	104.65 (18.03)
	**Blue**			**Yellow**		
	**L**	**a**	**b**	**L**	**a**	**b**
Uncalibrated	13.61 (2.71)	14.22 (2.94)	14.65 (2.20)	12.90 (1.96)	17.31 (2.51)	19.75 (3.31)
Linear regression	49.17 (4.73)	78.28 (7.31)	310.77 (29.33)	163.72 (6.69)	204.02 (9.87)	83.26 (6.39)
Polynomial regression	**7.65 (0.74)**	**9.99 (2.06)**	**7.93 (1.05)**	**3.77 (1.48)**	**4.10 (1.24)**	**8.91 (1.61)**
HPPCC-WCM	60.56 (7.88)	49.43 (7.61)	51.42 (9.26)	14.48 (16.56)	65.89 (7.41)	252.45 (16.67)
	**Gray**					
	**L**	**a**	**b**			
Uncalibrated	8.98 (1.95)	9.48 (1.09)	15.71 (3.96)			
Linear regression	117.32 (9.34)	257.94 (8.92)	180.36 (8.27)			
Polynomial regression	**5.67 (1.42)**	**8.39 (2.04)**	**8.14 (1.89)**			
HPPCC-WCM	22.82 (4.22)	23.65 (3.92)	52.87 (4.54)			

**Table 11:** ΔE_00_ of the considered methods for the chromatic clusters. The bold font highlights the best values throughout.

	Red	Green	Blue	Yellow	Gray
Uncalibrated	55.45 (6.21)	211.41 (22.39)	201.16 (19.35)	18.74 (4.56)	45.29 (6.86)
Linear regression	110.13 (7.47)	161.11 (5.69)	129.01 (8.07)	153.30 (8.51)	146.29 (6.94)
Polynomial regression	**35.12 (3.67)**	558.33 (44.10)	186.45 (13.96)	**5.83 (0.67)**	61.26 (5.90)
HPPCC-WCM	69.11 (7.23)	**68.03 (5.50)**	**79.12 (6.16)**	56.71 (5.15)	51.81 (5.28)

**Table 12:** Δ of the considered methods for the chemical clusters. The bold font highlights the best values throughout.

	**Lead**	**Iron**	**Copper**	**Copper (Organic)**	**Organic**	**Iron + Mn + Co**
Uncalibrated	21.14 (4.82)	20.64 (5.96)	26.89 (5.17)	29.49 (5.25)	44.53 (11.72)	22.64 (5.75)
Linear regression	290.97 (13.67)	112.50 (8.02)	939.86 (22.37)	188.19 (9.89)	304.21 (35.28)	131.05 (10.07)
Polynomial regression	**9.67 (1.22)**	**8.21 (1.94)**	**11.32 (1.16)**	**22.77 (1.92)**	**19.18 (5.92)**	**12.53 (2.47)**
HPPCC-WCM	136.13 (13.64)	212.49 (12.88)	211.75 (16.36)	233.65 (9.37)	226.56 (18.37)	193.52 (9.53)

**Table 13:** Δ of the considered methods for the chromatic clusters. The bold font highlights the best values throughout.

	Red	Green	Blue	Yellow	Gray
Uncalibrated	22.94 (4.35)	23.51 (5.01)	24.54 (4.02)	29.26 (5.19)	20.43 (5.25)
Linear regression	161.08 (13.49)	170.87 (9.13)	324.23 (18.08)	274.52 (13.35)	335.90 (16.27)
Polynomial regression	**13.14 (1.58)**	**14.78 (2.37)**	**14.87 (2.79)**	**10.51 (2.03)**	**12.99 (2.49)**
HPPCC-WCM	222.33 (13.35)	172.64 (8.35)	93.57 (8.26)	261.31 (11.31)	62.25 (5.78)

## Data Availability

Not applicable.

## Appendix A

In this appendix, a table presenting the pigments’ descriptions is provided.

**Table A1:** List of the available pigments with their name, chemical composition, and chemical and chromatic classes.

Tile	Pigment Name	Pigment Color	Chemical Composition	Chemical Class	Chromatic Class
Tile 1	Lead White	Lead white	2PbCO_3_.Pb(OH)_2_	Lead	Gray
Tile 4	Calcium Carbonate	White	CaCO_3_	Other	Gray
Tile 5	Vine Black German	Black	Retouching color in aldehyde resin 81. Carbon with impurities of potassium and sodium ions	Organic	Gray
Tile 9	Azurite MP, sky-blue light	Azure	Cu_3_(CO_3_)_2_.(OH)_2_	Copper	Blue
Tile 13	Smalt	Blue	K_2_O.nSiO_2_ with the presence of cobalt	Cobalt	Blue
Tile 15	Orpiment	Yellow	As_4_S_6_	Other	Yellow
Tile 16	Realgar	Orange, yellow	As_4_S_4_	Other	Yellow
Tile 19	Yellow Ochre Iron Oxide	Yellow	α-FeO(OH) or K,Fe(SO4)2(OH)6 or γFeO(OH))	Iron	Yellow
Tile 20	Raw Sienna Italian	Raw sienna, yellow, brown	Fe_2_O_3_.nH_2_O + MnO_2_ + Al_2_O_3_ + SiO_2_	Iron	Yellow
Tile 21	Massicot Litharge	Yellow	PbO	Lead	Yellow
Tile 22	Burnt Sienna Italian	Burnt sienna, red, brown	Fe_2_O_3_.nH_2_O + Al_2_O_3_ (60%) + MnO_2_ (1%)	Iron	Red
Tile 23	Burnt Umber Reddish	Burnt umber, brown	Fe_2_O_3_ + MnO_2_ + Si + Al_2_O_3_	Iron	Red
Tile 25	French Ochre SOFOROUGE	Red	SiO_2_ + Al_2_O_3_ + Fe_2_O_3_	Iron	Red
Tile 28	Pozzuolana Red Earth	Purple, red	Mix of lands	Iron	Red
Tile 30	Madder Lake Genuine	Pink, red	Organic nature	Organic	Red
Tile 31	Red Ochre English	Red	Fe_2_O_3_.nH_2_O	Iron	Red
Tile 33	Red Bole	Brown, red	Al_2_Si_2_O_5_(OH)_4_	Other	Red
Tile 34	Red Lead, minimum	Orange, red	PbO_4_	Lead	Red
Tile 36	Malachite Natural Standard	Green	Cu_3_(CO_3_).(OH)_2_	Copper	Green
Tile 41	Verdigris, synthetic	Blue, turquoise, green	Cu(CH_3_COO)_2_	Copper (organic)	Blue
Tile 42	Barium Sulfate	White	BaSO_4_	Other	Gray
Tile 43	Sepia Fine	Black-brown	Sepia, fine (colorant of cuttlefish)	Organic	Gray
Tile 44	Bone Black	Black	15–20% of carbon, 60–70% of Ca_3_(PO_4_)_2_	Other	Gray
Tile 45	Asphaltum Black	Black	High molecular weight hydrocarbons	Organic	Gray
Tile 46	Blue Bice	Blue, turquoise	Cu_2_(CO_3_)2.Cu(OH)_2_	Copper	Blue
Tile 47	Lapis Lazuli	Blue	(Na,Ca)_8_(AlSiO_4_)_6_ + % of iron	Iron	Blue
Tile 48	Ultramarine Ash	Blue ultramarine	Na_2_O_3_Al_6_SiO_2_.2Na_2_S	Other	Blue
Tile 49	Lead Tin Yellow Light	Lemon yellow	Lead stannate, type I (Pb_2_SnO_4_)	Lead	Yellow
Tile 50	Indian Yellow Imitation	Indian yellow	Consisting primarily of euxanthic acid salts	Organic	Yellow
Tile 51	Naples Yellow, dark	Naples yellow	Pb_2_Sb_2_O_7_	Lead	Yellow
Tile 52	Van Dyck Brown	Van Dyke brown	Consists mainly of humic acids and iron oxide	Iron	Red
Tile 53	Natural Cinnabar	Orange, red	Mineral cinnabar, HgS	Other	Red
Tile 54	Lac Dye	Pink, red	Lac dye (from coccus lacta secretion, Natural Red 25; gum lac, Indian lake)	Organic	Red
Tile 55	Vermilion	Vermilion red	Mine cinnabar, HgS	Other	Red
Tile 56	Caput Mortuum Reddish	Red, violet	Fe_2_O_3_	Iron	Red
Tile 57	Caput Mortuum Violet	Brown, violet	Fe_2_O_3_	Iron	Red
Tile 58	Green Earth Light	Green	Iron-based silicate	Iron	Green
Tile 59	Prussian Blue	Prussian blue	Fe_4_[Fe(CN)_6_]_3_.6H_2_O or KFe[Fe(CN)_6_].6H_2_O	Iron	Blue
Tile 60	Lead Tin Yellow II	Lemon yellow	Type II, Pb(Sn,Si)O_3_	Lead	Yellow
Tile 61	Naples Yellow from Paris	Yellow	Pb(Sb,Sn)O_3_	Lead	Yellow
Tile 62	Venetian Red	Venetian red	Fe_2_O_3_	Iron	Red
Tile 67	Lead Sulfate	White	PbSO_4_	Lead	Gray
Tile 68	Lithopone	White	BaSO_4_ + ZnS	Other	Gray
Tile 69	Titanium White Rutile	Titanium white	TiO_2_	Other	Gray
Tile 70	Zinc White	Zinc white	ZnO + % of iron	Iron	Gray
Tile 71	Zinc Sulfide	White	ZnS	Other	Gray
Tile 72	Manganese Black	Black	(Fe,Mn)_3_O_4_	Iron	Gray
Tile 73	Ploss Blue	Blue, turquoise,	(CuCa)CO_3_(CH_3_COO)_2_.2H_2_O)	Copper (organic)	Blue
Tile 74	Blue Verditer	Blue	CuCO_3_ Cu(OH)_2_	Copper	Blue
Tile 75	Ultramarine Blue very dark	Ultramarine blue	Al_6_Na_8_O_24_S_3_Si_6_	Other	Blue
Tile 76	Copper Blue	Blue, turquoise	Copper based	Copper	Blue
Tile 77	Zirconium cerulean blue	Cerulean blue	Derived from zircon	Other	Blue
Tile 78	Cavansite	Blue, turquoise	Ca(VO)Si_4_O_10_.4(H_2_O)	Other	Blue
Tile 79	Ultramarine Blue Dark	Ultramarine blue	Na_2_O_3_Al_6_SiO_2_.2Na_2_S	Other	Blue
Tile 80	Cobalt Blue Dark	Blue	(Co,Zn)_2_SiO_4_	Cobalt	Blue
Tile 81	Cobalt Blue Pale	Blue	CoAl_2_O_4_	Other	Blue
Tile 82	Natural Chromium Yellow or crocoite	Yellow	PbCrO_4_	Lead	Yellow
Tile 83	Cadmium Yellow n°6 medium	Cadmium yellow	CdS + ZnO	Other	Yellow
Tile 84	Permanent Yellow medium	Yellow	Organic nature	Organic	Yellow
Tile 85	Brilliant Yellow	Yellow	C_18_H_18_N_4_O_6_	Organic	Yellow
Tile 86	Studio Yellow	Yellow	C_16_H_12_Cl_2_N_4_O_4_	Organic	Yellow
Tile 87	Cobalt Yellow	Yellow	[Co(NO_2_)_6_]K_3_ + 3H_2_O	Cobalt	Yellow
Tile 88	Bismuth-Vanadate YelLow Lemon	Yellow	(Bi,V)O_4_	Other	Yellow
Tile 89	Baryte Yellow	Yellow	BaCrO_4_	Other	Yellow
Tile 90	Studio Pigment Yellow	Yellow	C_18_H_18_N_4_O_6_	Organic	Yellow
Tile 91	Studio Pigment Yellow Sun Gold	Yellow	Organic nature	Organic	Yellow
Tile 92	Cadmium Orange n°0 very light	Orange	Cd_2_SSe	Other	Red
Tile 93	Paliotol^®^ Orange	Orange	C_8_H_9_N	Organic	Red
Tile 94	Paliogen^®^ Orange	Orange	C_23_H_8_C_l8_N_4_O_2_	Organic	Red
Tile 95	Irgazin^®^ Yellow, light orange	Yellow	C_8_H_7_NO	Organic	Yellow
Tile 96	Isoindolol Orange	Orange	C_8_H_7_N	Organic	Red
Tile 97	Titanium Orange	Orange, yellow	Ti-Sb-Cr-O Rutile	Other	Yellow
Tile 98	Iron Oxide Orange 960	Orange	Fe(O)OH + Fe_2_O_3_	Iron	Red
Tile 99	IWA-Enogu^®^ Shinsia	Pink	Sodium aluminosilicate with oxides of metals other than iron	Other	Red
Tile 100	IWA-Enogu^®^ Iwamomo	Pink	Sodium aluminosilicate with oxides of metals other than iron	Other	Red
Tile 101	IWA-Enogu^®^ Usukuchi-Murasaki	Violet	Sodium aluminosilicate with oxides of metals other than iron	Other	Red
Tile 102	Côte d’Azur Violet	Gray, violet	Fe_2_O_3_	Iron	Gray
Tile 103	Thioindigo Red Lightfast	Red	Organic nature	Organic	Red
Tile 104	Cinquasia^®^ Violet RT 201 D	Reddish violet	Organic nature	Organic	Red
Tile 105	Ultramarine Violet medium	Violet, bluish	Sodium, alumino, sulfo, silicate	Other	Blue
Tile 106	Manganese Violet	Manganese violet	(NH_4_)_2_Mn_2_(P_2_O_7_)	Manganese	Blue
Tile 107	Cobalt Violet Dark	Cobalt violet	Co_3_(PO_4_)_2_	Cobalt	Blue
Tile 108	Pink color	Pink, red	Ca(Sn,Cr)SiO_5_	Other	Red
Tile 109	Cadmium Red n°2 medium	Cadmium red	CdS	Other	Red
Tile 110	Irgazine^®^ Scarlet DPP EK	Scarlet red	C_6_H_2_N_2_O_2_	Organic	Red
Tile 111	Alizarine Crimson dark	Pink, red	Colorant/organic pigment for coatings	Organic	Red
Tile 112	XSL Irgazine^®^ Red DPP	Red	Organic nature	Organic	Red
Tile 113	Rosso Sartorius	Brown, red	Fe_2_O_3_.nH_2_O	Iron	Red
Tile 114	Aegirine Fine	Green	NaFeSi_2_O_6_	Iron	Green
Tile 115	Andeer Green Fine	Green	Granite	Other	Green
Tile 116	Phthalo Green dark	Green, bluish	Cu(C_32_N_8_Cl_14_).16HCl	Copper (organic)	Green
Tile 117	Chromite	Green	FeCr_2_O_4_	Iron	Green
Tile 118	Cobalt Green	Green	Co_2_SnO_4_	Cobalt	Green
Tile 119	Cobalt Green Bluish A	Green, turquoise	Cobalt-based	Cobalt	Green
Tile 120	Chrome Oxide Green	Green	Cr_2_O_3_	Other	Green
Tile 122	Permanent Green	Green, turquoise	CoAl_2_O_4_	Cobalt	Green
Tile 123	Cadmium Green Light	Green	Cadmium-based	Other	Green
Tile 124	Cadmium Green Dark	Green	Cadmium-based	Other	Green
Tile 125	Fluorescent Pigment Blue	Blue	Unspecified	Other	Blue
Tile 126	Phthalo Blue	Blue	C_32_H_16_CuN_8_	Copper (organic)	Blue
Tile 127	Phthalo Blue Royal Blue	Blue	C_32_H_16_CuN_8_	Copper (organic)	Blue
Tile 129	Indanthren^®^ Blue	Blue	Unspecified	Organic	Blue
Tile 130	XSL Phthalo Blue Royal Blue Very Lightfast	Blue	C_32_H_16_CuN_8_	Copper (organic)	Blue
Tile 131	Indigo Blue Lake	Blue	Organic nature	Organic	Blue
Tile 132	Indigo Red-Violet	Blue, violet	Organic nature	Organic	Blue
Tile 133	Studio Pigment Sky Blue	Blue	Unspecified	Other	Blue
Tile 134	Studio Pigment Dark Blue	Blue	Unspecified	Other	Blue
Tile 135	XSL Translucent Yellow	Yellow	Unspecified	Iron	Yellow
Tile 136	IWA-Enogu^®^ Iwabeni	Red	Unspecified	Other	Red
Tile 137	Phthalo Green, yellowish	green, yellowish	Copper-based	Copper (organic)	Green
Tile 138	Heliogen^®^ Green	Green, bluish	Copper-based	Copper (organic)	Green
Tile A	Madder Lake glazing over natural cinnabar	Red	Organic nature	Organic	Red
Tile B	Azurite glazing over Madder Lake	Blue	Cu_3_(CO_3_)_2_.(OH)_2_	Copper	Blue
Tile C	Lapis Lazuli glazing over Azurite	Blue	(Na,Ca)_8_(AlSiO_4_)_6_ + % of iron	Iron	Blue
Tile D	Copper resinate glazing over Verdigris	Green	Cu(CH_3_CO)_2_.2Cu(OH)_2_.nH_2_O	Copper (organic)	Green
Tile E	Bisso (mixture of Madder Lake and Lapis Lazuli)	Blue	Organic nature	Organic	Blue
Tile F	Mixture of Azurite and Lead Tin Yellow Light	Green	Cu_3_(CO_3_)_2_^.^(OH)_2_ and Pb_2_SnO_4_	Copper lead (*)	Green

(*): Tile F presents both copper and lead in its chemical composition, so it belongs to both copper and lead clusters.
